# OCTET does not demonstrate a lack of effectiveness for community treatment orders

**DOI:** 10.1192/pb.bp.113.044800

**Published:** 2014-02

**Authors:** David Curtis

**Affiliations:** 1 University College London

## Abstract

In the Oxford Community Treatment Order Evaluation Trial (OCTET), patients were randomised either to be made subject to a community treatment order (CTO) or to be managed with Section 17 leave and discharge. No differences in outcome between the two groups were observed. Here it is argued that the patients studied were not those who might have benefited from a CTO and that the psychiatrists involved were unlikely to have used the provisions of a CTO assertively. Consideration of the lengths of time for which both Section 17 leave and CTOs were used supports the notion that CTOs were not used appropriately for a group of patients who might have benefited from them. Hence the results of this study should not be taken to provide any evidence as to the effectiveness or otherwise of CTOs.

The Oxford Community Treatment Order Evaluation Trial, known as OCTET and prominently reported in *The Lancet*, found no difference in outcome between patients randomised to be made subject to a community treatment order (CTO) or to be managed with Section 17 leave and discharge.^1^ The authors concluded: ‘In well coordinated mental health services the imposition of compulsory supervision does not reduce the rate of readmission of psychotic patients’. An accompanying commentary^2^ declares that ‘the case for urgent review of this legislation, both at government level and within the professions involved in CTO use, is now strong’, although it does then recommend sensible alternative methods to better assess the effect of CTOs. In a subsequent issue of *The Lancet*, correspondence was published drawing attention to some, but not all, of the problems of the study, but this will not have achieved the same prominence as the original article.^3–7^

In fact, the results of OCTET by no means support the authors' conclusions and indeed the study design itself meant that the hypothesis of effectiveness of CTOs, or a lack of it, could not be properly tested. Within the report the authors do express some of the relevant caveats and recognise problems with the design and execution of the study. However, they then make bold statements which are not borne out by an objective consideration of the results and which are potentially damaging to patient care, both nationally and internationally. The use of compulsory treatment in the community is controversial and it would be unfortunate if the findings of this study were misinterpreted as to cast doubt on its effectiveness.

There are a number of reasons why OCTET cannot throw light on the effectiveness of CTOs and these reasons are to some extent related to each other. Broadly, the concerns relate to the patients included, the clinicians who participated and the mode of implementation of the CTOs. Finally, there are concerns about the general attitudes of the researchers, and possibly of the participating clinicians as well, to the use of compulsory treatment.

## Attitudes to compulsory treatment

The researchers rightly note that there is wide variation of practice regarding the use of CTOs and, more widely, of the powers provided by the Mental Health Act 1983. However, in several places the wording used raises questions about the researchers' own attitudes. To take one example, they say that participants were randomised ‘to be discharged from hospital either on CTO or Section 17 leave’. But a patient who has been granted Section 17 leave has not been discharged. He has been allowed home on a temporary basis and remains subject to recall for further hospital treatment. Elsewhere they write of using Section 17 ‘for some hours or days, or even exceptionally weeks’ and that it is ‘a well established rehabilitation practice, used for brief periods’. Most strikingly, they write, ‘Patients can be discharged directly from Section 3 without the need for either Section 17 leave or a CTO, and most are.’

The final claim seems to be plainly incorrect. It would be practically unheard of for a patient to be discharged directly from detention under Section 3 without any use of Section 17 leave and it is certainly not the case that ‘most are’. The other statements give the impression that the researchers' view of Section 17 leave is that it might be used briefly following a period of detention as a form of trial of discharge arrangements before proceeding, all being well, to actual discharge. It is true that some psychiatrists do only use Section 17 leave briefly in this way but there are others who implemented a quite different approach, at least before CTOs became available. This approach would have been to allow the patient home on ‘extended leave’ for months at a time, with acceptance of treatment as a condition of continuing leave. Indeed, some psychiatrists would intermittently recall such patients to hospital for the purpose of having their period of detention under Section 3 extended (as the renewal could only be carried out while the patient was in hospital), resulting in *de facto* compulsory community treatment. This practice was eventually deemed to be unlawful but nevertheless some psychiatrists would continue to manage patients in the community on Section 17 leave for periods of several months until the Section 3 expired. For some patients such an arrangement could continue for up to a year. If during the period of leave there were problems necessitating recall to hospital, then, if the section were close to expiring, the psychiatrist could take the opportunity to renew the section prior to a further period of leave.

Such a use of extended Section 17 leave is not without its critics. However, it is undeniable that some psychiatrists have been happy to use it in this way and they would argue that it has enabled their patients to remain fairly well outside of hospital for long periods of time. The point is that the authors of the paper, who say that it might be used ‘exceptionally’ for ‘weeks’, seem clearly not to share this outlook and actually fail even to acknowledge that such practices exist. This observation means that there are grounds for concern about the attitudes of at least some of the authors. One also wonders whether those psychiatrists who agreed to participate in the project had similar views and might have had a general distaste for assertively using the legal powers to enforce psychiatric treatment. That is, one might suspect that psychiatrists who strongly believed in the value of CTOs might have been reluctant to submit their patients for randomisation.

## Selection of patients

Although the randomisation is described as being between the use of CTO and Section 17 leave, it is clear that what is really meant is the use of CTO *v*. fairly brief Section 17 leave followed by discharge. (We can infer that only a brief use of Section 17 leave was intended both from the general outlook communicated by the study authors and from the fact that in practice patients were discharged after a median of only 8 days on leave.) Thus the only patients who might be considered suitable for randomisation would be those for whom discharge or CTO were thought to be equally suitable, and hence would not include patients for whom the provisions of a CTO were judged to be necessary. Even if one does not accept the point that the period of Section 17 leave was expected to be brief, it remains the case that the legal constraints would mean that it could not be continued for longer than a matter of months, and hence any patient expected to have a continuing requirement for compulsory treatment in the community could not have been considered eligible for randomisation.

Hence, it broadly seems that the only patients likely to be entered for OCTET randomisation were those for whom it is thought that the CTO would be unnecessary.

To emphasise this point, consider a patient who accepts depot medication while subject to Section 3 and on Section 17 leave, but who repeatedly refuses it and defaults from follow-up as soon as any period of compulsion under Section 3 ends. If such a pattern were established and if the patient presented with significant risk issues when unwell, then they would represent a perfect candidate for the use of a CTO, but they could not possibly have been selected for randomisation and included in this study.

This issue is somewhat muddied by the authors' report that they obtained a legal opinion that there was an area of equipoise between Section 17 and CTO regimes. This does indeed seem reasonable if one considers the situation at a single point in time. However, arguably the key difference between the two is the length of time which they can be applied for. For any patient who might need compulsory treatment for more than a few months the two regimes would obviously be substantially different and such a patient could not have been included in the randomisation process.

In general, it seems that the only patients who might have been eligible for inclusion in the study were those for whom one could make the judgement that a CTO was unlikely to be particularly useful.

## Selection of psychiatrists

As the authors point out, there is widespread variation in attitudes of psychiatrists towards compulsory treatment. At one extreme are psychiatrists who are reluctant to deprive patients of autonomy and who will use compulsory measures as sparingly as possible, even if they recognise that this may lead to more distress or poorer functioning for the patient, at least in the short term. At the other end are psychiatrists who will take a more paternalistic approach and who will enthusiastically use the powers provided by the Mental Health Act to impose on patients' treatment plans which they regard as being in the best interests of the patient and the wider public, even if the patient might be vigorously opposed to them, at least in the short term.

Where CTOs are concerned, it is natural to assume that some psychiatrists would regard them as providing a long-awaited opportunity to ensure that patients would continue to receive treatment in the community while others would regard them as an unacceptable intrusion and as a violation of their patients' human rights. A spectrum in between would consist of psychiatrists with a range of different attitudes and expectations.

Arguably, the nature of the study meant that the psychiatrists who volunteered to participate would be largely those who were doubtful of the value of CTOs and hence would be happy to submit patients for randomisation, with the risk that they would end up being subject to Section 17 for only a limited period of time. It would be reasonable to assume that such psychiatrists would also tend to be more reluctant to intervene assertively if a patient who had been placed on a CTO were to show early signs of deterioration and/or non-compliance.

Thus, the study design might be expected to recruit psychiatrists who were ambivalent about the usefulness of CTOs as well as patients for whom the usefulness of a CTO was deemed to be unclear.

However, there is an additional dimension regarding the psychiatrists involved which is worthy of separate consideration. This is that in at least some cases the psychiatrist instituting the CTO would not be the same as the one responsible for implementing it. The authors report that almost all of the collaborating services underwent reorganisation, sometimes resulting in the separation of in-patient and out-patient services, and that many patients were passed to psychiatrists unfamiliar with the trial. The authors report a large number of protocol violations, where the patient was not in fact provided with the intervention to which they had been randomised, and they attribute these in part to such changes in psychiatrist. They report that a sensitivity analysis omitting protocol violations did not alter the findings. Nevertheless, setting the protocol violations aside, it seems clear that for many patients the psychiatrist agreeing to randomisation was not the same as the psychiatrist implementing the CTO. It is not difficult to imagine that a community psychiatrist, perhaps unfamiliar with CTOs and perhaps unenthusiastic about them, might not represent an ideal candidate to test their usefulness. Of course, there is a widely held view that the split between in-patient and out-patient psychiatry is of itself a powerful contributor to the difficulties of maintaining patients in the community. The so-called ‘functional split’ means that the community psychiatrist is working with a patient who has been acutely unwell in hospital and who has been made subject to a treatment plan which somebody else has instituted. A perverse incentive exists, which is that if the patient gets admitted again then the community psychiatrist will no longer have to look after them and will no longer be responsible for any adverse events which might befall the patient or those in contact with the patient. It is not unreasonable to suspect that such considerations could have had an impact on the degree to which the responsible psychiatrist in the community made full use of the available provisions of CTOs to do everything possible to prevent the need for readmission.

## OCTET in practice

I have argued that there could be *a priori* grounds to suspect that OCTET might not have constituted a fair test of the effectiveness of CTOs. The study might have tended to incorporate psychiatrists who were dubious about their value and who would have selected only patients who could be managed equally well with or without a CTO. Such patients might then have been looked after in the community by other psychiatrists who might be even less enthusiastic about CTOs in general and who might in fact have had little interest in putting in the necessary effort to see that the maximum value was gained from their use. Is there any evidence to support this view? I believe that there is.

First, I would draw attention to the extremely short time for which Section 17 leave was used. The number of days for which patients were subject to Section 17 leave is stated as a median 8 and an interquartile range (IQR) of 0–37, with a mean of 45.5 and a standard deviation of 80.7. So half of all patients randomised to the Section 17 arm were fully discharged after just over a week on leave. And a quarter never had any leave at all (though, admittedly, a few of these may be patients who were randomised but then never left hospital). Many psychiatrists would find it very difficult to understand that there could be a patient who could be included in the study because their clinical condition was such that a CTO was ‘necessary’ while, having been randomised to the Section 17 arm, after the very briefest trial of leave or after no leave at all they could be completely discharged.

The fact that the mean is so much higher than the median implies that there were a handful of patients who were kept on Section 17 leave for a long period of time. However, the fact that the top of the IQR is stated to be 37 means that three-quarters of patients were discharged after little more than a month of trial leave. Again, it is hard to see that these are the patients which many psychiatrists would envisage as being appropriately managed with a CTO.

Second, we can look at the length of time for which the CTO itself was used in patients who were randomised to this management plan. Here, the median number of days is given as 183 with the mean being roughly the same, 170.1. The IQR is given as 0–299. The first thing to say is that it is difficult to believe that these figures are actually correct. The IQR starting at zero would imply that a quarter of patients spent no time at all subject to any legal constraint in the community. This could either be because they were never in fact discharged or because they were discharged but (as a protocol violation) without any use being made of either a CTO or Section 17 leave. It does seem somewhat unlikely that such a high proportion of patients would have been dealt with in this way, but if these figures are accurate then they do emphasise how few patients were in reality managed in accordance with the randomised allocation.

The mean and median periods for which patients in the CTO arm were subjected to compulsory community treatment were about 6 months. Section 17 leave can run for variable amounts of time but a CTO would typically be applied for distinct periods of 6 months at a time. Shorter periods might be brought about by discharges directed by tribunals, by readmission or by protocol violations involving the use of Section 17 leave instead. The authors state: ‘The median duration of the initial CTOs in our trial was 6 months, indicating that about half were being renewed’. This seems to represent a fundamental misunderstanding of the interpretation of a sample median. Given that many participants will have had an identical value for the quantity under consideration, here the number of days on compulsory community treatment, it is not the case that half of participants will have had a value higher than this. In this context, all the median tells us is that this was the middle rank. We know that it cannot be the case that half of the patients were subject to a CTO for 6 months and half for 12 months, because if so, the mean period would be 9 months, whereas in reality it was slightly under 6. The higher value of the IQR of 299 implies that only a quarter of participants were subject to a CTO for longer than 10 months. The fact that the median period was 183 does not imply that half of patients were subjected to a CTO for longer than 183 days, but that a substantial number of participants were detained for exactly 183 days, i.e. 6 months. Speaking approximately, the summary statistics reported would be correct if a quarter of patients were never in fact subject to a CTO, half were subject to one period of 6 months and a quarter had the CTO extended for a second period, meaning that it continued for the 12-month follow-up period of the study. This quarter-half-quarter pattern would much better fit with the results reported and would in fact mean that only a third, not half, of patients who were originally placed on a CTO then had it extended.

Having claimed that half of CTOs were renewed, the authors then say that this is an indicator that they were used appropriately. Regardless of whether the true proportion was a third or a half, I believe that many psychiatrists would strongly disagree with the judgement that this represented appropriate usage. There is a view that the true value of a CTO is to encourage a patient's compliance with a treatment plan over an extended period of time. From such a perspective, the use of a CTO for ‘only’ 6 months would not be appropriate. If one only wanted to ensure compliance for a few months then logically one could simply use the provisions of Section 17 leave. Thus these figures provide another strong pointer to the conclusion that the patients randomised in this study were not those for whom a CTO might be expected to offer benefit.

## Conclusions

There are grounds to suspect that OCTET was based on a group of patients for whom there was no real indication for the use of a CTO, and that they might have been managed by psychiatrists who had little enthusiasm to utilise the provisions of a CTO assertively over an extended period of time to do everything possible to support patients to remain in the community. It is undeniable that a very important group of patients were completely excluded from the study – those for whom discharge could not even be contemplated if a CTO were not available as a management option.

It is a matter of some concern that the authors of the study in fact acknowledge at least some of these reservations but then draw conclusions which are unjustified. They themselves allude to the clinicians and patients who were not part of the study because arguments in favour of the use of CTOs were so strong that randomisation was precluded. But then they write: ‘The evidence is now strong that the use of CTOs does not confer early patient benefits despite substantial curtailment of individual freedoms’. Additionally, they write in the abstract: ‘In well coordinated mental health services the imposition of compulsory supervision does not reduce the rate of readmission of psychotic patients’.

I believe that neither conclusion is justified by the findings of this study. I believe that a more appropriate conclusion would be that it remains a strong possibility that a small subgroup of patients, characterised by repeated relapses secondary to non-compliance and with only partial insight, might derive enormous benefit from more prolonged periods of compulsory treatment in the community and might be supported in building full and productive lives outside hospital.

Whether or not CTOs are effective could not have been determined by OCTET as it was designed. The effectiveness of legislation and policy are difficult or impossible to evaluate using the kind of randomised clinical trial which may be appropriate for individual components of treatment, such as antipsychotic medication. I am not aware of any clinical trial which has demonstrated that detention and treatment under Section 3 of the Mental Health Act is effective, and if one were to attempt such a trial one could envisage that it would likewise produce negative results, because the patients for whom it would be most useful would be excluded from randomisation. The accompanying commentary suggests that evidence might instead be sought through the large-scale collation and analysis of routine data,^2^ and this does indeed seem a more promising approach. In any event, the findings from OCTET should not be interpreted as telling us anything conclusive about the effectiveness or otherwise of compulsory treatment in the community.
